# 非小细胞肺癌肺转移？抑或第二（多）原发肺癌——影响诊疗策略的重要概念

**DOI:** 10.3779/j.issn.1009-3419.2014.07.03

**Published:** 2014-07-20

**Authors:** 克能 陈

**Affiliations:** 100142 北京，北京大学肿瘤医院暨恶性肿瘤发病机制及转化研究教育部重点实验室胸外一科 Key Laboratory of Carcinogenesis and Translational Research (Ministry of Education), Department Ⅰ of Thoracic Surgery, Peking University Cancer Hospital, Beijing 100142, China

随着年代的不同，非小细胞肺癌（non-small cell lung cancer, NSCLC）的临床特征及诊疗方式已经发生了巨大的变化，表现为从中心型病变向周围型病变的转变、鳞癌向腺癌的转变、巨块型向小结节的转变、单发病灶向多发病灶的转变。NSCLC的治疗也从单一学科治疗向多学科治疗转变，外科治疗从常规开胸向微创精准外科转变，放疗也从简单放疗向立体定向精确放疗转变（stereotactic body radiation therapy, SBRT），内科治疗更是步入了靶向时代，与之相适应的则是概念的更新。其中对“小结节病灶”、“第二原发癌/转移癌”的认识是这些众多变化的核心内容，是影响着治疗策略的重要概念更新。本文就影响治疗策略的肺癌肺转移及多发性肺癌的研究进展作一综述。

## 1 对NSCLC患者主病灶以外“小结节”的考虑

虽然目前没有任何针对NSCLC患者余肺组织“微小结节”（＜5 mm）及“小结节”（＞5 mm）是转移癌，抑或是所谓第二原发癌的前瞻性研究。但已有许多其他临床试验结果可供借鉴。首先，必须认识到随着高分辨率薄层计算机断层扫描（computed tomography, CT）扫描的不断改善，检出肺部“微小”结节及“小”结节的几率大大增加，这不但出现在针对肺癌的健康普查人群中，也同样出现在被诊断为肺癌的患者以及肺癌治疗后的长期存活人群中。但因对小结节的认识概念不尽相同，医生及患者的思想有相当的混乱，常导致过度检查甚至过度治疗，抑或治疗不足。2012年NLTS研究^[1]^经过对5万多例人群的普查确立了低剂量螺旋CT用以肺癌高危人群普查可降低肺癌死亡率20%。然而，在CT组26, 309例受试人群中共检出6, 561个结节中，肺癌仅占1%（270个），其余均为良性结节。更有甚者为了明确是否为肺癌的病理诊断导致的相关死亡率高达6.1/10, 000。

因此，有必要了解一下目前对“小”结节的认识。2005年Fleischner影像学会即推荐8 mm以下肺内小结节的处理原则^[2]^。根据年龄、吸烟史及既往恶性肿瘤病史等因素分为低危组与高危组。若病灶≤4 mm，低危组患者无需随诊，高危组患者需12个月后复查胸部CT，若病灶无变化则无需进一步随诊；若病灶为4 mm-6 mm，低危组患者需12个月后复查胸部CT，若病灶无变化也无需进一步随诊，对高危组患者初始6个月-12个月复查胸部CT，若病灶无变化则改为18个月-24个月复查。但对于磨玻璃结节影（ground-glass nodule, GGO）或半GGO需要更长时间随诊以除外“惰性”腺癌可能；若病灶直径为6 mm-8 mm，对低危组患者初始6个月-12个月复查胸部CT，若病灶无变化则改为18个月-24个月复查，高危组患者初始3个月-6个月复查胸部CT，若病灶无变化则改为9个月-12个月及24个月复查；若病灶直径＞8 mm，低危及高危组均需3个月、9个月及24个月分别复查胸部CT，或正电子发射体层摄影术（positron emission tomography, PET）扫描及/或活检。

2013年Fleischner影像学会再次更新了推荐指南^[3]^对于孤立性纯GGO若直径≤5 mm无需进行CT随诊，直径＞5 mm需要3个月后复查胸部CT评估，若没有变化每年随诊一次并且连续3年；孤立性部分实性结节，起初需要3个月后复查胸部CT，若实性成分＜5 mm，则每年随诊一次并且连续3年，若实性成分增大≥5 mm则建议活检或手术切除；多发性纯磨玻璃结节影若直径≤5 mm，需每2年-4年复查胸部CT；若直径＞5 mm但无实性成分，需要3个月后复查胸部CT评估，若没有变化每年随诊一次并且连续3年；若主要病灶伴部分实性或实性成分，需要3个月后复查胸部CT，若没有变化尤其病灶直径＞5 mm，则建议活检或手术切除。

不难看出，对肺部“小”结节的认识包括诊断与治疗，都在趋于保守。2011年肺腺癌的多学科新分类也释放了同样的信息，影像学上检出的某些结节或许是癌前病变或原位癌，呼吁新的治疗策略问世。因此对NSCLC患者同时或异时出现在其余肺组织的“小”结节，首先应进行良恶性，惰性与非惰性的甄别，其次再考虑是否需要活检与手术。当然，这仍然需要更好的更有针对性的临床证据支持与验证。

## 2 对NSCLC患者第二原发癌的认识

在出现Martini & Melamed标准之前，对已诊断有NSCLC的同时其他肺组织在CT上又有“小”病灶者多被诊断为“转移”，对NSCLC治疗后甚至治愈后的异时性出现在CT上的“小”病灶往往也被诊断为“复发”。无论是同时诊断为“转移”还是异时诊断为“复发”，多认为系晚期不可治愈性疾病。其治疗策略也多以姑息治疗为主，使患者的生存大大折扣。鉴于大量事实证明这些所谓的同时性“转移”与异时性“复发”，其临床预后与转归远好于传统意义上的“晚期”患者，不得不使人们考虑有到其他可能性的存在，这就是Martini & Melamed标准产生的背景。

Martini & Melamed标准^[4]^的主要精髓有二。其一，凡是两个病灶组织学类型不一样时，无论何种情况，均应诊断为独立的“第二（多）原发肺癌”；其二，如果组织学类型一样，应该说第二原发癌只适合早期癌，首先要排除远处转移与N2、N3（甚至N1），其次解剖学上有各自不同的位置（段、叶、肺），最后各病灶的生长方式（或分化程度）要不一样，如肺腺癌能够看出从癌前病变到原位癌直至实体癌的发展过程。当然Antakli等^[5]^尝试将DNA倍体分析结果补充到Martini & Melamed标准中，但是由于检测方法费时并且缺乏敏感性，未在临床推广。也有人尝试在分子水平鉴别转移癌与第二原发癌。Girard等^[6]^对多原发肺癌病例进行了分子生物学及临床病理学研究，应用比较基因杂交技术鉴别转移瘤或第二原发癌。基因组分析结果与临床病理诊断不一致率达18%，认为表皮生长因子受体（epidermal growth factor receptor, *EGFR*）/*Kras*基因突变检测有助于鉴别肺内转移瘤或第二原发肺癌。Ono等^[7]^探究了p53、p16、p27及c-erbB2在50例多原发肺癌、20例肺内转移瘤及30例淋巴结转移病例中的免疫组化表达差异，认为这些指标可作为多原发肺癌临床诊断与分期的重要指标。

## 3 区别第二原发NSCLC和NSCLC肺转移的意义

区别第二原发NSCLC和NSCLC肺转移的最主要原因是两者预后差异极大，第二原发癌的疗效明显好于转移癌的疗效，既便是对侧“小结节”为转移性NSCLC，在第7版TNM（tumor-node-metastasis）分期也作了区别对待，首次将远处转移M分为M1a（对侧肺转移）与M1b（除肺内转移外的远处转移）。两者的1年及5年生存率存在巨大差异，分别为46%、3%及22%、1%[危险比（hazard ratio, HR）=1.56，*P*＜0.000, 1）]^[8]^。其实，当一侧确诊为NSCLC而对侧出现“小结节”的鉴别诊断（M1a或第二原发癌）仍非常困难，必须严格按照Martini & Melamed标准加以区别。为此，ACCP 2013年复习了既往研究，显示同时性第二原发癌切除后的平均5年生存率可达33%，最高者可达61%（20篇文献，*n*=1, 221）；异时性第二原发癌手术治疗的平均5年生存率达41%，最高者可达77%（15篇文献，*n*=814）；当第二原发癌在影像学表现为GGO或伴GGO时，切除后平均5年生存率高达84%，最高者达100%（7篇文献，*n*=263）。

即使同时性被诊断为转移者，按新版TNM分期，分别归为T3（同叶）其切除后平均5年生存率也可达37%，最高为66%（共19篇文献，*n*=1, 068），或T4（同侧不同叶）切除后平均5年生存率为19%，最高为31%（共8篇文献，*n*=353），但对侧“小结节”（M1a）的研究资料非常少见，仅有来自SEER、CCR、IASLC质量不高的回顾性数据，5年生存率降至3%左右，提示需要积累更多的资料。因此，认真区别NSCLC肺转移与第二原发癌十分重要。

## 4 认识NSCLC肺转移的重要性

早在1829年人们就已认识到恶性肿瘤的重要特征之一是转移^[9]^。1889年由Paget^[10]^提出并沿用至今的有关转移的基本理论“土壤与种子”学说，奠定了人体某些器官如脑、肺、肝、骨是恶性肿瘤的好发转移器官，随着研究的进展，人们也进一步认识到在这些常见转移靶器官的原发肿瘤来源的顺序也各有不同。2008年Chiang等^[11]^经过大量数据分析，对常见肿瘤靶器官的原发肿瘤来源做了总结，认为脑转移癌的常见转移来源依次为肺癌、乳腺癌、恶性黑色素瘤、肾癌和结直肠癌^[12]^；肺转移癌的常见转移来源依次为肾癌、结直肠癌、恶性黑色素瘤、乳腺癌和肉瘤^[13, 14]^；肝转移癌的常见转移来源依次为结直肠癌、胰腺癌、乳腺癌、肺癌和肾癌^[15]^；骨转移癌的常见转移来源依次为乳腺癌、肺癌、前列腺癌、肾癌和结直肠癌^[16]^。毋庸置疑，肺是各种恶性肿瘤的最常见靶转移器官之一。不难理解，NSCLC作为最常见高度恶性肿瘤，在就诊时就有约50%的患者出现转移^[17]^，因此，肺癌肺转移是临床不能忽视的重要问题之一。

## 5 寻找NSCLC转移的特殊类型——寡转移状态

自1933年全肺切除成功治疗肺癌以来^[18]^，肺癌的治疗历经了许多重要的阶段，如1968年出现的NSCLC与SCLC不同的诊断治疗策略；1977年出现的按解剖学受累范围划分的肺癌TNM分期系统^[19]^；20世纪80年代就已认识到NSCLC“孤立性脑转移”经适当治疗后可长期生存，使我们对转移的认识从“一旦转移即是绝对晚期（Ⅳ期），是不可治愈转的疾病”向“孤立性转移治疗后能长期生存”的概念转变。随着对转移认识的加深，由Hellman与Weichselbaum^[20]^在1995年提出了更进一步的新概念即“寡转移状态”，该理论认为NSCLC出现从隐匿性转移发展成为致命的广泛转移之间存在一段相当长的稳定时期，在这段时间转移癌因原发癌的不同、转移靶器官的不同、转移数量的不同，而有不同的稳定时间、不同的预后，为个体化综合考虑患者的治疗方案提供了依据。为此，2013年Niibe在综合了大量研究后给出了“寡转移状态”的综合考虑因素及临床预后风险度。作者认为良好预后者包括：异时性NSCLC发生于脑和/或肾上腺2个以内的转移灶；结直肠癌发生于肺和/或肝2个以内的转移灶；肾癌发生于任何器官2个以内的转移灶。中度风险偏好的类型包括异时性乳腺癌发生于肝和/或骨和/或肺2个以内的转移灶；SCLC发生于脑2个以内的转移病灶；NSCLC发生于脑和/或肾上腺3个-5个的转移灶；结直肠癌发生于肺和/或肝3个-5个的转移灶；肾癌发生于任何器官3个-5个的转移灶；同时性NSCLC发生于脑和/或肾上腺2个以内的转移灶；结直肠癌发生于肺和/或肝2个以内的转移灶；肾癌发生于任何器官2个以内的转移灶。中度风险偏坏包括：异时性乳腺癌发生于肝和/或骨和/或肺3个-5个的转移灶；同时性NSCLC发生于脑和/或肾上腺3个-5个的转移灶；结直肠癌发生于肺和/或肝3个-5个的转移灶；乳腺癌发生于肝和/或骨和/或肺3个-5个的转移灶。预后差的转移则包括：胰腺癌、恶性黑色素瘤、肉瘤发生于全身任何部位任何数量的转移，以及无论原发癌如何，也无论转移至何种器官，只要转移灶的个数≥5个，即表明预后不良。虽然文章未涉及NSCLC肺转移，但不难想象NSCLC肺转移也有寡转移状态，如何区别并加以适当治疗从而延长生存是重要的临床课题。

总之，当一个病灶确诊为NSCLC时，在同时或异时出现在CT上的位于余肺组织上的“微小”结节与“小”结节，首先应鉴别良性与恶性，恶性时要区别第二（多）原发与转移，对于转移癌要区别是寡转移还是广泛转移。只有这样，才能确保患者不会失去可治愈的机会。从事胸部肿瘤学的临床工作者应对此概念及时更新并积极投身到更多的临床研究中，为NSCLC患者提供更好的治疗。
